# How Well Do Low Population-Specific Values for Muscle Parameters Associate with Indices of Poor Physical Health? Cross-Sectional Data from the Geelong Osteoporosis Study

**DOI:** 10.3390/jcm11102906

**Published:** 2022-05-20

**Authors:** Sophia X. Sui, Kara L. Holloway-Kew, Natalie K. Hyde, Lana J. Williams, Monica C. Tembo, Emma West, Julie A. Pasco

**Affiliations:** 1 IMPACT—Institute for Mental and Physical Health and Clinical Translation, Deakin University, Geelong, VIC 3220, Australia; k.holloway@deakin.edu.au (K.L.H.-K.); natalie.hyde@deakin.edu.au (N.K.H.); l.williams@deakin.edu.au (L.J.W.); m.tembo@deakin.edu.au (M.C.T.); westem@deakin.edu.au (E.W.); julie.pasco@deakin.edu.au (J.A.P.); 2 Department of Medicine–Western Campus, The University of Melbourne, St Albans, VIC 3010, Australia; 3 Department of Epidemiology and Preventive Medicine, Monash University, Melbourne, VIC 3800, Australia; 4 Barwon Health, University Hospital Geelong, Geelong, VIC 3220, Australia

**Keywords:** sarcopenia, indices of poor physical health, cut-offs, muscle

## Abstract

We aimed to examine associations between skeletal muscle deficits and indices of poor health. Cut-points for skeletal muscle deficits were derived using data from the Geelong Osteoporosis Study and definitions from the revised European Consensus on Definition and Diagnosis and the Foundation for the National Institutes of Health. Participants (*n* = 665; 323 women) aged 60–96 year had handgrip strength measured by dynamometry and appendicular lean mass by whole-body dual-energy X-ray absorptiometry. Physical performance was assessed using the Timed Up and Go test. Sex-specific cut-points were equivalent to two standard deviations below the mean young reference range from the Geelong Osteoporosis Study. Indices of poor health included fractures, falls, and hospitalisations. Low trauma fractures since age 50 year (excluding skull, face, digits) were self-reported and confirmed using radiological reports. Falls (≥1 in the past 12 months) and hospitalisations (past month) were self-reported. Logistic regression models (age- and sex-adjusted) were used to examine associations. Receiver Operating Characteristic curves were applied to determine optimal cut-points for handgrip strength, Timed Up and Go, appendicular lean mass/height^2^, and appendicular lean mass/body mass index that discriminated poor health outcomes. There were 48 participants (6.9%) with hospitalisations, 94 (13.4%) with fractures, and 177 (25.3%) with at least one fall (≥1). For all cut-points, low handgrip strength was consistently associated with falls. There was little evidence to support an association between low appendicular lean mass, using any cut-point, and indices of poor health. Optimal cut-offs for predicting falls (≥1) were: handgrip strength 17.5 kg for women and 33.5 kg for men; Timed Up and Go 8.6 s for women and 9.9 s for men; appendicular lean mass/height^2^ 6.2 kg/m^2^ for women and 7.46 kg/m^2^ for men; and appendicular lean mass/body mass index 0.6 m^2^ for women and 0.9 m^2^ for men. In conclusion, muscle strength and function performed better than lean mass to indicate poor health. These findings add to the growing evidence base to inform decisions regarding the selection of skeletal muscle parameters and their optimal cut-points for identifying sarcopenia.

## 1. Introduction

Sarcopenia is defined as reduced muscle mass and function with ageing [1,2]. Sarcopenia is considered a component of physical frailty in as much as it shares domains and measurements with frailty, in particular poor physical function and muscle strength [3,4,5,6]. Currently, there is no universally accepted operational definition for sarcopenia [3]. Further, there is confusion regarding inconsistencies in the thresholds applied to identify low muscle mass, muscle strength, and poor muscle function [3]. The most widely employed definitions are those from the European Working Group on Sarcopenia in Older People (EWGSOP) [1] and the Foundation for the National Institutes of Health (FNIH) in the USA [2]. We have reported that prevalence estimates for sarcopenia vary substantially in different geographic areas and also in homogeneous samples when different criteria and cut-off points are applied [7,8]. Nevertheless, despite these differences, sarcopenia and its components have consistently been associated with physical limitations [9], mental ill-health [10], and poor cognitive function [11,12].

Age-related or disease-induced health conditions, such as chronic disease, sensory and motor deficits, and physical and mental frailty, are associated with poor performance in walking, climbing, bending, and carrying [13]. These adverse health conditions are also associated with detrimental outcomes (including falls, fractures, and hospitalisations) that have significant impacts on social well-being [13,14,15] and quality of life [16,17,18]. Few studies have examined the association between the components of sarcopenia using population-specific values and indices of poor health [19]. Thus, we aimed to examine associations between measures of skeletal muscle deficits and indices of poor health, using cut-points that identify low values in the distribution of the same population.

## 2. Methods

### 2.1. Study Design

This cross-sectional analysis involved data from the 15-year follow-up phases of the Geelong Osteoporosis Study (GOS), collected from 2010 to 2014 for women and 2016 to 2019 for men. Detailed information about the GOS is published elsewhere [20]. Briefly, age-stratified samples of women and men were selected at random using the electoral roll as the sampling frame. In total, 1494 women were recruited from 1993–1997 (ages 20–93 years, 77% participation), and 1540 men were recruited from 2001–2006 (ages 20–96 years, 67% participation) and assessed at subsequent follow-up phases. The cohorts are essentially Caucasian (~98%).

### 2.2. Participants

A total of 894 women and 624 men participated in the 15-year follow-ups, respectively. Of these, 405 women and 347 men were aged ≥60 years old and included in this analysis. Of the remaining participants, 358 women and 341 men provided HGS data, 323 women and 342 men provided ALM data, and 328 women and 340 men provided TUG data. Thus, data for identifying sarcopenia were available for 323 women and 342 men aged ≥ 60 years (missing data: HGS men = 1; ALM women = 35; TUG women = 30, men = 2).

The study was approved by the Barwon Health Human Research Ethics Committee. Written informed consent was obtained from all participants.

### 2.3. Data

#### 2.3.1. Anthropometry

Weight and height were measured to the nearest ±0.1 kg and ±0.01 m, and body mass index (BMI) was calculated as weight/height^2^ (kg/m^2^).

#### 2.3.2. Muscle strength

Handgrip strength (HGS, kg) was measured using an analogue hand-held dynamometer (Jamar, Sammons Preston, Bolingbrook, IL, USA) for women and an electronic hand-held dynamometer for men (Vernier, LoggerPro3). Trained researchers explained and demonstrated the testing procedure to each participant before measurement trials. With the participant seated in a comfortable position and the arm holding the dynamometer flexed at the elbow to 90 degrees, the participant was asked to squeeze the device as hard as possible for several seconds, and the peak reading was recorded. This procedure was repeated for each hand, and there was no time interval between trials. For men, the protocol was similar, except there was a 5-s interval between trials. Maximum values were used for analysis. HGS values measured with the Vernier device were transformed to Jamar equivalent values according to the following equation:

HGSJamar (kg) = 9.50 + 0.818 × HGSVernier (kg) + 8.80 × Sex

where sex = 1 for men, that was developed by measuring maximum HGS on each device for 45 men and women aged 21–67 years, as previously described [8].

#### 2.3.3. Timed up and Go Test

Timed Up and Go (TUG), a test of mobility that involves static and dynamic balance [21], involved measuring the time taken (in seconds) by the participant to stand up from a chair (without armrests), walk to a marked line (3 m distance), turn around then return to the chair and sit down.

#### 2.3.4. Lean Mass

Lean mass, a proxy measure for muscle mass, was obtained from whole-body dual-energy X-ray absorptiometry (DXA; Lunar Prodigy-Pro, Madison, WI, USA); appendicular lean mass (ALM) was equivalent to the sum of lean mass for the arms and legs. ALM was expressed relative to height as ALM/height^2^ (kg/m^2^) and BMI as ALM/BMI (m^2^).

### 2.4. Population-Specific Values for Sarcopenia Components

Population-specific cut-points were determined as equivalent to two standard deviations (SDs) below sex-specific mean values for young reference groups (ages ≤ 49 years) generated from the same population [22,23,24,25]. For women, the cut-point for low HGS was <16 kg [24] and, for men, <31 kg [8]. Low lean mass was identified as ALM/height^2^ <5.3 kg/m^2^ for women and <6.94 kg/m^2^ for men [22], low ALM/BMI as <0.512 m^2^ for women and <0.827 m^2^ for men [23]. Using the same approach, we calculated that the cut-off for low TUG was >9.3 s for women and >9.9 s for men.

### 2.5. Indices of Poor Health

Fractures (excluding fractures of the skull, face, fingers, and toes as well as those occurring from high trauma) were ascertained by self-report and confirmed using radiological reports. Fractures were included in the analyses if there was at least one incident fracture since the age of 50 years. There were more fractures than participants with fractures because some participants had sustained more than one fracture. Falls in the past 12 months and hospitalisations in the past month were self-reported. For fractures, falls, and hospitalisations, at least one (in each case) was considered an index of poor health. We used the Research Electronic Data Capture tool (hosted by Barwon Health) to collect and manage these data [26].

### 2.6. Statistical Analysis

Analyses were conducted using IBM SPSS (V24, USA) and Minitab (v18, USA). The data were visually checked for normality using histograms. Logistic regression models were developed to investigate the association between sarcopenia parameters and the indices of poor health. The dependent variable (falls, fractures, and hospitalisations) corresponds to odd ratios of the independent variable (sarcopenia parameters using GOS population-specific, EWGSOP2, and FNIH cut-offs). Potential covariates tested in the models included age and sex. Interaction terms were checked in the final models.

Furthermore, we estimated cut-points for HGS, TUG, ALM/height^2^, and ALM/BMI that best discriminated the presence or absence of indices of poor health. Similar to our previous study, the location of each optimal cut-point was determined by the principle that the sensitivity and specificity are closest to the value of the area under the Receiver Operating Characteristic (ROC) curve, and the absolute value of the difference between the sensitivity and specificity is the smallest [27].

## 3. Results

### 3.1. Participant Characteristics

Table 1 shows the participant characteristics. Participants’ ages ranged from 60 to 96 years, and the mean BMI was in the overweight category. There were 177 (25.3%) participants with at least one fall and 38 (5.4%) with two or more falls over the past 12 months. There were 94 who had fractures since the age of 50 years (rib 14, wrist 13, ankle 9, humerus 8, spine 7, foot 7, forearm 6, patella 6, shoulder 6, tibia 6, hand 5, hip 4, clavicle 3, femur 3, fibula 3). There were 48 (6.9%) participants who had been hospitalised during the previous month.

### 3.2. Associations between Low Muscle Parameters and Indices of Poor Health

Table 2 shows odds ratios (OR) and 95% confidence intervals (95% CIs) for the associations between low muscle parameters and indices of poor health before and after adjusting for age and sex. For all cut-points, low HGS was consistently associated with falls (≥1). Low HGS was associated with hospitalisation when using EWGSOP2 criteria. There was little evidence to support an association between ALM, using any cut-point, and indices of poor health. No interaction terms were detected in any models.

### 3.3. Cut-Offs of Skeletal Muscle Deficits Obtained Using Receiver Operating Characteristic Curves

The cut-off for HGS that best predicted falls (≥1) was 17.5 kg for women (sensitivity 0.86, specificity 0.35; area under the curve: 0.62 95% CI 0.55–0.69; *p* < 0.001); and 33.5 kg for men (sensitivity 0.72, specificity 0.44; area under the curve: 0.60 95% CI 0.52–0.67; *p* = 0.02). The TUG test cut-off was 8.6 s for women (sensitivity 0.73, specificity 0.38; area under the curve: 0.61 95% CI 0.54–0.68; *p* = 0.001); and 9.9 s for men (sensitivity 0.59, specificity 0.70; area under the curve: 0.69 95% CI 0.62–0.77; *p* < 0.001). ALM/height^2^ was 6.20 kg/m^2^ for women (sensitivity 0.74, specificity 0.43; area under the ROC curve: 0.55 95% CI 0.48–0.62; *p* = 0.18); and 7.46 kg/m^2^ for men (sensitivity 0.86, specificity 0.27; area under the curve: 0.55 95% CI 0.47–0.63; *p* = 0.25); ALM/BMI was <0.61 m^2^ for women (sensitivity 0.43, specificity 0.72; area under the curve: 0.56 95% CI 0.50–0.63; *p* = 0.07); 0.91 m^2^ for men (sensitivity 0.46, specificity 0.72; area under the curve: 0.60 95% CI 0.53–0.68; *p* = 0.009).

## 4. Discussion

In this study, we report associations between measures of skeletal muscle deficits and indices of poor health, using different cut-points to identify low values. Overall, muscle strength performed better than lean mass measures for indicating poor indices of health. In particular, HGS and TUG were consistently associated with falls, regardless of the cut-offs applied. Lean mass measures were not associated with falls, fractures, or hospitalisations. Further, we determined optimal point estimates for skeletal muscle deficits that identify indices of poor health, including falls, fractures, and hospitalisation.

The current study found that HGS, regardless of cut-off, is a predictor of falls. Using data from 353 men and 245 women aged 65–98 years from the same study, we have previously reported that sarcopenic obesity was associated with an increased risk of falls and emphasised that physical performance substantially contributed to the identified associations [28]. Similarly, a recent study from Turkey also reported that individuals with sarcopenic obesity had a higher risk for falls compared to those without sarcopenic obesity [29]. The cross-sectional study involved 423 men and women aged ≥65 years, and sarcopenia was categorised according to EWGSOP1 using population-specific (Turkish) cut-off points. Body composition parameters were assessed using a bioelectrical impedance analyser. A recent cross-sectional Australian study by Kirk et al. examined the association between sarcopenia and health indicators in older individuals [19]. This study included 356 people (median age 79 years, 24.8% men) from the community. Sarcopenia was defined using low HGS and gait speed cut-offs recommended by Sarcopenia Definitions and Outcomes Consortium. The health outcomes included falls, fractures (past 5 years), malnutrition, depression, balance confidence, fear of falling, static balance, dynamic balance, and body composition. The study reported that sarcopenia was positively associated with malnutrition, depression, fear of falling as well as recurrent falls and fractures and negatively associated with poor balance confidence. In our current study, sarcopenia parameters were not observed to be associated with fractures. The point estimates for fractures seem to suggest a pattern of poor muscle and higher fracture risk; however, there could be less statistical power to detect the significant relationships. A systematic review and meta-analysis [30] reported similar results that sarcopenia was associated with a higher rate of falls and a higher incidence of hospitalisations; however, it was unclear whether sarcopenia was associated with the incidence of fractures. This review focused on longitudinal studies (range of follow-up: 3 months to 9.8 years), and only studies that used the EWGSOP1 criteria to diagnose sarcopenia were included.

Another systematic review and meta-analysis, including nine prospective studies, examined associations between sarcopenia and the risk of fractures among community-dwelling older adults [31]. The sarcopenia definitions considered were the EWGSOP, FNIH, International Working Group for Sarcopenia (IWGS), and Asian Working Group for Sarcopenia (AWGS). An association between sarcopenia and fractures was observed only for men when applying the AWGS definition [31]. Another systematic review and meta-analysis included five cohort studies with a total of 27,990 participants [32], showing that sarcopenia was associated with incident fracture.

In the current study, only HGS using EWGSOP2 was associated with hospitalisation. In contrast, a systematic literature review reported that sarcopenia predicted hospitalisation in older women regardless of the specific population or the sarcopenia definition used [33]. This review included a total of 2832 participants in five studies and demonstrated that sarcopenia was associated with hospitalisation in community and hospital settings in both cross-sectional and longitudinal studies. It is possible that our study was underpowered to detect such an association.

A systematic literature review assessed associations between sarcopenia with falls and fractures among older adults [34]. Thirty-six studies (52,838 individuals) were included in the systematic review, while thirty-three studies (45,926 individuals) were included in the meta-analysis. This article revealed that people with sarcopenia had a higher risk of falls and fractures compared with those without sarcopenia, which was independent of study design, population, sex, sarcopenia definition, continent, and study quality.

The TUG test was included as a component of sarcopenia in EWGSOP2. In our study, we consistently found that a poor TUG test time was associated with falls. This confirms the TUG test as a predictor for falls. Since the TUG test was devised in 1991, reports of optimal cut-offs for increased fall risk have ranged from 10 to 33 s [35,36,37]. A recent study (2021) [38] that evaluated the reliability and validity of the TUG test to predict falls in the elderly included 148 participants aged 60–90+ years, of whom 58 reported a fall in the previous year. Similar to our study, optimal TUG cut-points for predicting falls were determined using ROC curves. As the calculated cut-points increased for increasing age groups, the authors suggested that age-specific cut-points should be used.

Given the nature of the dataset utilised, this study was able to apply both population-specific cut-offs and criteria recommended by the EWGSOP and FNIH. The current cross-sectional study minimised the variance that arises naturally from differing participant characteristics in multiple samples. Falls and hospitalisations were self-reported, which may have been affected by recall bias. However, although we relied on some self-reported data, fractures were confirmed using radiological reports.

In conclusion, muscle strength and performance performed better than lean mass measures for indicating poor indices of health. These findings add to the growing evidence base to inform decisions regarding the selection of skeletal muscle parameters and their optimal cut-points for identifying sarcopenia.

## Figures and Tables

**Table 1:** Participant characteristics. Data are expressed as mean (±SD) or median (IQR) or *n* (%).

	Total	Women	Men
	***N* = 665**	***N* = 323**	***N* = 342**
Age (year)	71 (65–78)	70 (64–75)	70.0 (66–78)
**Anthropometry**			
Weight (kg)	78.9 (±15.7)	73.9 (±15.4)	83.9 (±13.8)
Height (m)	1.66 (±0.09)	1.59 (±0.06)	1.73 (±0.07)
BMI (kg/m^2^)	28.6 (±5.2)	29.1 (±6.0)	28.1 (±4.1)
**Sarcopenia components**			
HGS (kg)	29 (±1.0)	23 (±6)	37 (±6)
ALM/height^2^ (kg/m^2^)	7.44 (±1.20)	6.59 (±0.79)	8.25 (±0.93)
ALM/BMI (m^2^)	0.74 (±0.19)	0.59 (±0.10)	0.89 (±0.12)
TUG (s)	9.2 (8.0–10.9)	9.1 (7.9–10.8)	9.2 (8.0–10.7)
**Indices of poor health**			
Hospitalisation (≥1 in past month)	48 (6.9%)	23 (6.5%)	25 (7.4%)
Fracture (≥1 since age 50 year)	94 (13.4%)	58 (16.2%)	36 (10.5%)
Falls (≥1 in the past 12 month)	177 (25.3%)	110 (30.7%)	67 (19.6%)

HGS: handgrip strength; ALM: appendicular lean mass; ALM/BMI: appendicular lean mass/body mass index; TUG: Timed Up and Go (TUG) test.

**Table 2:** Odds ratios (OR 95% confidence interval) of binary logistic models for the associations between low muscle parameters and indices of poor health.

		Unadjusted		Adjusted for Age and Sex	
Sarcopenia Indicators (Predictors)	Indices of Poor Health (Outcomes)	Odds Ratios (95% CI)	*p* Value	Odds Ratios (95% CI)	*p* Value
**ALM/BMI_GOS_**					
	Hospitalisation (≥1 in past month)	1.23 (0.64–2.36)	0.54	1.10 (0.56–2.16)	0.79
	Fracture (≥1 since age 50 year)	1.17 (0.72–1.93)	0.53	1.17 (0.70–1.95)	0.54
	Falls (≥1 in the past 12 month)	**1.51 (1.03–2.22)**	**0.03**	1.43 (0.96–2.14)	0.08
**ALM/height^2^_GOS_**					
	Hospitalisation (≥1 in past month)	0.82 (0.19–3.54)	0.79	0.68 (0.16–3.02)	0.62
	Fracture (≥1 since age 50 year)	1.64 (0.49–5.48)	0.42	1.73 (0.51–5.87)	0.38
	Falls (≥1 in the past 12 month)	1.43 (0.68–2.98)	0.35	1.22 (0.57–2.63)	0.61
**HGS_GOS_**					
	Hospitalisation (≥1 in past month)	1.86 (0.99–3.49)	0.05	1.54 (0.76–3.10)	0.23
	Fracture (≥1 since age 50 year)	1.36 (0.82–2.25)	0.24	1.17 (0.67–2.04)	0.59
	Falls (≥1 in the past 12 month)	**2.44 (1.66–3.58)**	**<0.001**	**2.04 (1.33–3.14)**	**0.001**
**TUG_GOS_**					
	Hospitalisation (≥1 in past month)	0.73 (0.39–1.34)	0.30	0.89 (0.44–1.80)	0.74
	Fracture (≥1 since age 50 year)	**0.56 (0.35–0.88)**	**0.01**	0.63 (0.38–1.05)	0.08
	Falls (in the past 12 months)	**0.14 (0.29–0.59)**	**<0.001**	**0.58 (0.38–0.87)**	**0.008**
**ALM/height^2^_EWGSOP2_**					
	Hospitalisation (≥1 in past month)	1.18 (0.41–3.45)	0.76	1.02 (0.34–3.04)	0.97
	Fractures (≥1 since age 50 year)	1.26 (0.57–2.78)	0.57	1.15 (0.51–2.58)	0.73
	Falls (≥1 in the past 12 month)	1.44 (0.77–2.67)	0.25	1.14 (0.60–2.18)	0.69
**HGS_EWGSOP2_**					
	Hospitalisation (≥1 in past month)	**3.28 (1.54–7.00)**	**0.002**	**3.23 (1.35–7.78)**	**0.009**
	Fracture (≥1 since age 50 year)	1.95 (1.01–3.77)	0.05	1.38 (0.67–2.84)	0.39
	Falls (in the past 12 month)	**3.24 (1.88–5.57)**	**<0.001**	**1.87 (1.03–3.38)**	**0.04**
**TUG_EWGSOP2_**					
	Hospitalisation (≥1 in past month)	0.59 (0.13–2.64)	0.59	0.80 (0.17–3.74)	0.77
	Fracture (≥1 since age 50 year)	055 (0.18–1.70)	0.30	0.67 (0.21–2.16)	0.50
	Falls (≥1 in the past 12 month)	**0.14 (0.05–0.37)**	**<0.001**	**0.20 (0.07–0.56)**	**0.002**
**ALM/BMI_FNIH_**					
	Hospitalisation (≥1 in past month)	1.36 (0.68–2.71)	0.38	1.25 (0.62–2.53)	0.53
	Fractures (≥1 since age 50 year)	1.20 (0.71–2.04)	0.50	1.12 (0.65–1.92)	0.68
	Falls (≥1 in the past 12 month)	1.38 (0.91–2.08)	0.13	1.17 (0.77–1.80)	0.46
**HGS_FNIH_**					
	Hospitalisation (≥1 in past month)	**2.55 (1.13–5.77)**	**0.02**	2.37 (0.93–6.01)	0.07
	Fractures (≥1 since age 50 year)	**2.10 (1.08–4.07)**	**0.03**	1.47 (0.71–3.05)	0.28
	Falls (≥1 in the past 12 month)	**3.34 (1.92–5.82)**	**<0.001**	**1.90 (1.03–3.48)**	**0.04**

GOS: Geelong Osteoporosis Study; HGS: handgrip strength; ALM: appendicular lean mass; ALM/BMI: appendicular lean mass/body mass index; TUG: Timed Up and Go (TUG); EWGSOP2_:_ the European Working Group on Sarcopenia in Older People (revised); FNIH: the Foundation for the National Institutes of Health.

## Data Availability

The data presented in this study are available on request from the corresponding author.
